# Self-replenishment cycles generate a threshold response

**DOI:** 10.1038/s41598-019-53589-1

**Published:** 2019-11-20

**Authors:** Hiroyuki Kurata

**Affiliations:** 1 Department of Bioscience and Bioinformatics, Kyushu Institute of Technology, Fukuoka, Japan; 2 Biomedical Informatics R&D Center, Kyushu Institute of Technology, Fukuoka, Japan

**Keywords:** Biophysics, Computational biology and bioinformatics, Systems biology

## Abstract

Many metabolic cycles, including the tricarboxylic acid cycle, glyoxylate cycle, Calvin cycle, urea cycle, coenzyme recycling, and substrate cycles, are well known to catabolize and anabolize different metabolites for efficient energy and mass conversion. In terms of stoichiometric structure, this study explicitly identifies two types of metabolic cycles. One is the well-known, elementary cycle that converts multiple substrates into different products and recycles one of the products as a substrate, where the recycled substrate is supplied from the outside to run the cycle. The other is the self-replenishment cycle that merges multiple substrates into two or multiple identical products and reuses one of the products as a substrate. The substrates are autonomously supplied within the cycle. This study first defines the self-replenishment cycles that many scientists have overlooked despite its functional importance. Theoretical analysis has revealed the design principle of the self-replenishment cycle that presents a threshold response without any bistability nor cooperativity. To verify the principle, three detailed kinetic models of self-replenishment cycles embedded in an *E. coli* metabolic system were simulated. They presented the threshold response or digital switch-like function that steeply shift metabolic status.

## Introduction

To analyze or design a biochemical network, it is critically important to understand fundamental relationships between network structure and function, or design principles by which elementary networks, called building blocks and network motifs, generate a variety of biological functions^1–3^. The elementary network can be regarded as the minimal or small subnetwork responsible for particular functions such as ultrasensitivity, amplification, adaptation, noise filtration, pulse generation, oscillation, and bistability. As well as LEGO blocks, the elementary networks can be assembled in a hierarchical manner to synthesize a large-scale network with a high-level or complex function. An understanding of the elementary networks and their combined networks provides insight into a rational guidance for how a robust biological circuit is synthesized to carry out a specific function. A database of such biological networks^4^ was presented to cover a broad range of elementary networks, as well as their derivatives and combined networks.

Metabolic cycles are fundamental structures in a cellular system, including the tricarboxylic acid (TCA) cycle, glyoxylate (GX) cycle, Calvin cycle, urea cycle, carnitine cycle, substrate cycles^5^, and coenzyme (ATP, NADH, NADPH, FADH, etc.) recycling^6–8^ (Table 1). Those critical cycles are well-known to catabolize and anabolize different metabolites for efficient energy and mass conversion. On the other hand, the substrate cycles and coenzyme recycling have been intensively studied for an understanding of their particular functions^7,9–12^.

**Table 1:** Examples of metabolic cycles.

Elementary cycle	
TCA cycle	AcCoA + **OAA**— > ICIT
	ICIT— > **OAA** + 2 CO_2_
Urea cycle	CBP + **ORN** − > CITR
	CITR— > **ORN** + Urea
Calvin cycle	3 **RuBP** + 3 CO_2_ − > 6 PGA
	6 PGA— > 3 **RuBP** + G3P
Substrate cycle	**PYR** − > LAC (lactic dehydrogenase)
	LAC − > **PYR** (lactate oxidase)
Coenzyme recycling	Substrate1 + **NADH** − > Product1 + NAD^+^
	Substrate2 + NAD^+^ − > Product2 + **NADH**
**Self-replenishment cycle**	
GS-GOGAT cycle	NH_3_ + **GLU** − > GLN (GS)
	GLN + 2KG − > **2 GLU** (GOGAT)
	[overall reaction] NH_3_ + **GLU** + 2KG − > **2 GLU**
Glyoxylate (GX) cycle	AcCoA + **OAA**— > ICIT
	ICIT − > SUC + GX
	SUC— > **OAA**
	GX + AcCoA − > MAL— > **OAA**
	[overall reaction] 2 AcCoA + **OAA**— > **2 OAA**
Glucose PTS with glycolysis	GLC + **PEP** − > G6P + PYR (glucose PTS)
	G6P— > 2 G3P— > **2 PEP** (glycolysis)
	[overall reaction] GLC + **PEP**— > **2 PEP** + PYR

Bold metabolites are the substrates/products responsible for forming the cycle. The self-replenishment cycle doubles the substrate. Reaction pathways are simplified.

Substrate cycles often consist of two antagonist enzymes. One enzyme catalyzing a reaction is opposed by the other enzyme that catalyzes its opposite reaction^5,13–15^. Many substrate cycles are seen in metabolism, such as the L-lactic dehydrogenase-L-lactate oxidase (PYR-LAC) cycle^3^, alanine transaminase-L-glutamate oxidase (2KG-GLU) cycle^16^, malate dehydrogenase-malate oxidase (OAA-MAL) cycle^17^, and glutamine synthetase (GS)-glutamate synthase (GOGAT) (GLU-GLN) cycle^18^. Since the substrate cycles, which are typically coupled with coenzymes, consume chemical energy or reducing power, they were early called futile cycles^19^. At present, however, these substrate cycles are known to amplify or accelerate a response against a weak stimulus^5^. The amplifier function can be strengthened by coupling two substrate cycles so that one cycle supplies a substrate to the other cycle^17^ (Supplementary Table S1). A simple substrate cycle typically shows monostability, while it can show bistability or irreversibility when one enzyme of the two enzymes is subject to substrate inhibition or nonlinear regulation^15^. A substrate cycle with a zero-order reaction provides ultrasensitivity or a switching device (formate/lactic dehydrogenases model cycle)^20^.

Coenzyme recycling is ubiquitous and involved in many reactions to supply chemical energy or reducing power (Supplementary Table S1). Some coenzyme recycling cycles show an amplifier function in a similar manner to that of substrate cycles, because their reaction structures are the same as those of the substrate cycles. Coenzyme recycling cascades have been extensively studied that directly bind multiple metabolic reactions coupled with coenzymes^10,11,21^. These can be classified into two types: the coenzyme recycling cascade with conservation of coenzymes^8,10,22^ and those with accumulation of coenzymes, where the coenzymes are supplied from neighboring pathways^11^. The coenzyme recycling cascade with coenzyme conservation shows slow relaxation to the steady state in response to external changes and provides robust properties to external and internal fluctuation^10^. The coenzyme recycling cascade with accumulated coenzymes presents turbo design, where the accumulated coenzymes enhance the cascade reaction in a positive feedback manner. The turbo design is exemplified by the yeast glycolysis pathway that a catabolic pathway begins with an ATP-requiring activation step and the subsequent metabolism yields a surplus of ATP^11^.

**Figure 1:**
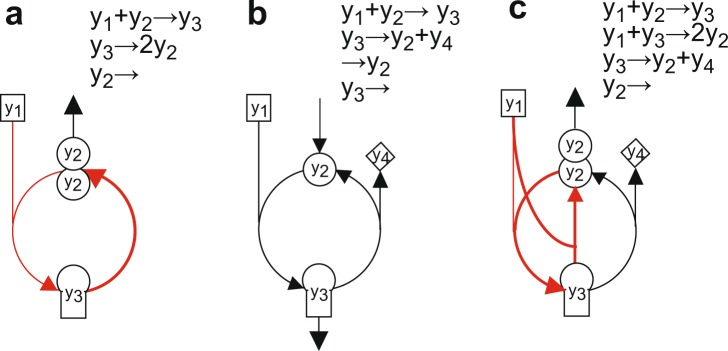
Network maps of metabolic cycles. (**a**) Self-replenishment cycle. (**b**) Elementary cycle. An external replenishment (anaplerotic) reaction is added. (**c**) Self-replenishment with elementary cycles. They are the simplified version of the self-replenishment (GX) cycle combined with the elementary (TCA) cycle. The self-replenishment cycle is colored in red.

From a stoichiometric standpoint, metabolic cycles can be divided into two types: the elementary cycle and the self-replenishment cycle (Table 1, Fig. 1ab, Supplementary Fig. S1). The elementary cycle is well-known to convert multiple substrates into different products and reuse one of the products as a substrate. The self-replenishment cycle converts different substrates into multiple or two identical products, which are autonomously supplied as the substrates within the cycle. This study tries to identify the self-replenishment cycle as a metabolic switch with threshold.

Design principles of such a biological switch have been extensively investigated. A shifted Michaelis-Menten relationship responds to substrate above a threshold but does not any response below the threshold. This threshold function was generated by multiple modifications^23^, suicide inhibitors^24,25^, or positive feedback loop^26^. As a characteristic threshold response, the rectified linear unit (ReLU) was proposed that combines a competitive inhibition with the conserved total level of substrate and inhibitors and a positive feedback loop, exemplified by yeast Spt-Ada-Gcn5-acetyltransferase (SAGA) histone acetylation complex^27^. It linearly responds to substrate above a threshold.

To date studies on metabolic cycles have focused on various functions such as amplification, acceleration and robustness, while they underexplored or overlooked any essential function of the self-replenishment cycles. The objective of this study is to first define the self-replenishment cycle and to theoretically reveal the design principle that presents a threshold response and all or nothing response. Three established, detailed kinetic models of *E. coli* are numerically simulated to verify the proposed design principle.

## Methods

### Definition of self-replenishment cycles

From a stoichiometric standpoint, metabolic cycles are classified into two types: the elementary cycle and self-replenishment cycle (Table 1). The elementary cycle is the ubiquitous cycle that converts multiple substrates into different products and reuses one of them as a substrate (Fig. 1b), exemplified by the TCA cycle, urea cycle, Calvin cycle, or coenzyme recycling. The cycle readily stops without any external replenishment (anaplerotic reaction) flux when the recycled products degrade with time (*v*_*r*_ > 0). To drive the cycle, substrates are typically supplied from the outside. On the other hand, the self-replenishment cycle (Fig. 1a) converts multiple substrates into two identical products and reuses them as substrates, where the substrates are autonomously supplied within the cycle. The self-replenishment cycle is exemplified by the GS-GOGAT cycle, the GX cycle, or the glucose PTS with glycolysis (Table 1). The GS-GOGAT cycle generates two glutamates from 2KG and glutamine. Intensive abstraction demonstrates that the GX cycle produces two OAAs from two AcCoAs and OAA and the glucose PTS with glycolysis doubles PEP. Those doubled products are fed back as the substrates to the cycle.

### Theoretical model

#### Self-replenishment cycle

Two molecules of *y*_1_ and *y*_2_ were merged into molecule y_3_ and then y_3_ is split into two identical molecules of *y*_2_, as shown in Fig. 1a. By assuming that the concentration of y_1_ is constant, $y(1)={y}_{1}^{0}({\rm{constant}})$, a theoretical model of the self-replenished cycle is given by:

1 $$\frac{dy(2)}{dt}=-\frac{{k}_{m}\cdot y(1)\cdot y(2)}{({K}_{m1}+y(1))({K}_{m2}+y(2))}+2\frac{{k}_{s}\cdot y(3)}{{K}_{s3}+y(3)}-{k}_{r}\cdot y(2)$$

2 $$\frac{dy(3)}{dt}=\frac{{k}_{m}\cdot y(1)\cdot y(2)}{({K}_{m1}+y(1))({K}_{m2}+y(2))}-\frac{{k}_{s}\cdot y(3)}{{K}_{s3}+y(3)}$$

where *y*(1), *y*(2) and *y*(3) are the molecular concentrations of y_1_, y_2_ and y_3_, respectively, *k*_*m*_ the merge (input) rate constant, *k*_*s*_ the split rate constant, *k*_*r*_ the removal (cataplerotic) rate constant, *k*_*m*1_ the Michaelis constant for *y*1 for the merge reaction, *k*_*m*2_ the Michaelis constant for *y*2 for the merge reaction, and *k*_*s*3_ the Michaelis constant for *y*3 for the split reaction. The steady state levels of *y*(2) and *y*(3) were obtained with respect to *k*_*s*_, as follows.

3 $${y}_{ss}(2)=\{\begin{array}{ll}0\,(stable) & \,{k}_{m}^{\ast }\le {k}_{r}{K}_{m2}\\ \frac{{k}_{m}^{\ast }}{{k}_{r}}-{K}_{m2} & \,{k}_{r}{K}_{m2} < {k}_{m}^{\ast } < {k}_{s}+{k}_{r}{K}_{m2}\\ NA & \,{k}_{m}^{\ast }\ge {k}_{s}+{k}_{r}{K}_{m2}\,\end{array}$$

4 $${y}_{ss}(3)=\{\begin{array}{ll}0\,(stable) & \,{k}_{m}^{\ast }\le {k}_{r}{K}_{m2}\\ \frac{{K}_{s3}({k}_{m}^{\ast }-{k}_{r}{K}_{m2})}{{k}_{s}-{k}_{m}^{\ast }+{k}_{r}{K}_{m2}} & \,{k}_{r}{K}_{m2} < {k}_{m}^{\ast } < {k}_{s}+{k}_{r}{K}_{m2}\\ NA & \,{k}_{m}^{\ast }\ge {k}_{s}+{k}_{r}{K}_{m2}\,\end{array}.$$

where

5 $${k}_{m}^{\ast }=\frac{{k}_{m}{y}_{1}^{0}}{{K}_{m1}+{y}_{1}^{0}}.$$

and *NA* indicates that no positive, steady-state solution exists. The flux of the self-replenishment cycle is given by:

6 $${v}_{self}=\frac{{k}_{s}\cdot y(3)}{{K}_{s3}+y(3)}$$

In the region of *NA*, indeed, *y*(3) diverges with time, while *y*(2) approaches a limit value. By setting

$$y(2)\to 0,\,\frac{dy(3)}{dt}\to \infty ,$$

the limit of *y*(2) is given by:

7 $${y}_{\mathrm{lim}}(2)=\frac{2{k}_{s}-{k}_{m}^{\ast }-{K}_{m2}{k}_{r}+\sqrt{{{k}_{r}}^{2}{{K}_{m2}}^{2}+4{k}_{s}{k}_{r}{K}_{m2}+2{k}_{m}^{\ast }{k}_{r}{K}_{m2}+4{{k}_{s}}^{2}-4{k}_{m}^{\ast }{k}_{s}+{k}_{m}^{\ast 2}}}{2{k}_{r}}.$$

### Elementary cycle

As a reference model, an elementary cycle with the external replenishment flux was built (Fig. 1b). By assuming $y(1)={y}_{1}^{0}({\rm{constant}})$, a theoretical model is given by

8 $$\frac{dy(2)}{dt}=-\frac{{k}_{m}\cdot y(1)\cdot y(2)}{({K}_{m1}+y(1))({K}_{m2}+y(2))}+\frac{{k}_{e}\cdot y(3)}{{K}_{e3}+y(3)}+{v}_{a}$$

9 $$\frac{dy(3)}{dt}=\frac{{k}_{m}\cdot y(1)\cdot y(2)}{({K}_{m1}+y(1))({K}_{m2}+y(2))}-\frac{{k}_{e}\cdot y(3)}{{K}_{e3}+y(3)}-{k}_{r}\cdot y(3)$$

where *v*_*a*_ is the external replenishment flux or called the anaplerotic reaction flux, *k*_*e*_ the conversion rate constant, and *k*_*e*3_ the Michaelis constant for *y*3 for the conversion reaction. Generally, the recycled products are supplied from the outside to run the cycle. The steady-state concentrations of *y*(2) and *y*(3) were obtained as

10 $${y}_{ss}(2)=\{\begin{array}{cl}NA & {k}_{m}^{\ast } < \frac{{{v}_{a}}^{2}+{k}_{e}{v}_{a}+{k}_{r}{v}_{a}{K}_{e3}}{{v}_{a}+{k}_{r}{K}_{e3}}\\ -\frac{{{v}_{a}}^{2}{K}_{m2}+{k}_{e}{v}_{a}{K}_{m2}+{k}_{r}{v}_{a}{K}_{m2}{K}_{e3}}{{k}_{e}{v}_{a}-{k}_{m}^{\ast }{v}_{a}+{{v}_{a}}^{2}-{k}_{m}^{\ast }{k}_{r}{K}_{e3}+{k}_{r}{v}_{a}{K}_{e3}} & {k}_{m}^{\ast }\ge \frac{{{v}_{a}}^{2}+{k}_{e}{v}_{a}+{k}_{r}{v}_{a}{K}_{e3}}{{v}_{a}+{k}_{r}{K}_{e3}}\end{array}$$

11 $${y}_{ss}(3)=\{\begin{array}{ll}NA & {k}_{m}^{\ast } < \frac{{{v}_{a}}^{2}+{k}_{e}{v}_{a}+{k}_{r}{v}_{a}{K}_{e3}}{{v}_{a}+{k}_{r}{K}_{e3}}\\ \frac{{v}_{a}}{{k}_{r}} & {k}_{m}^{\ast }\ge \frac{{{v}_{a}}^{2}+{k}_{e}{v}_{a}+{k}_{r}{v}_{a}{K}_{e3}}{{v}_{a}+{k}_{r}{K}_{e3}}\end{array}.$$

where *NA* means that there is no positive steady-state solution. The steady-state flux of the elementary cycle is given by

12 $${v}_{elem}=\frac{{k}_{e}{y}_{ss}(3)}{{K}_{e3}+{y}_{ss}(3)}$$

Indeed, *y*(2) diverges with time; *y*(3) approaches to a limit value. By setting

$$y(2)\to \infty ,\,\frac{dy(3)}{dt}\to 0,$$

the limit of *y*(3) is given by:

13 $${y}_{\mathrm{lim}}(3)=\frac{-{k}_{e}+{k}_{m}^{\ast }-{k}_{r}{K}_{e3}+\sqrt{{{k}_{r}}^{2}{{K}_{e3}}^{2}+2{k}_{e}{k}_{r}{K}_{e3}+2{k}_{m}^{\ast }{k}_{r}{K}_{e3}+{{k}_{e}}^{2}-2{k}_{m}^{\ast }{k}_{e}+{k}_{m}^{\ast 2}}}{2{k}_{r}}.$$

#### Self-replenishment and elementary cycles

The self-replenishment cycle (Fig. 1a) was combined with the elementary cycle (Fig. 1b, *v*_*a*_ = 0), as shown in Fig. 1c. Since the combined cycles can be regarded as simplified GX cycle and TCA cycles, they lead to an understanding of mechanisms by which how the GX cycle works together with the TCA cycle. By assuming $y(1)={y}_{1}^{0}({\rm{constant}})$, a theoretical model of the combined cycles was provided by

14 $$\frac{dy(2)}{dt}=-\frac{{k}_{m}\cdot y(1)\cdot y(2)}{({K}_{m1}+y(1))({K}_{m2}+y(2))}+\frac{{k}_{e}\cdot y(3)}{{K}_{e3}+y(3)}+2\frac{{k}_{s}\cdot y(1)\cdot y(3)}{({K}_{s1}+y(1))({K}_{s3}+y(3))}-{k}_{r}y(2)$$

15 $$\frac{dy(3)}{dt}=\frac{{k}_{m}\cdot y(1)\cdot y(2)}{({K}_{m1}+y(1))({K}_{m2}+y(2))}-\frac{{k}_{e}\cdot y(3)}{{K}_{e3}+y(3)}-\frac{{k}_{s}\cdot y(1)\cdot y(3)}{({K}_{s1}+y(1))({K}_{s3}+y(3))}$$

where *K*_*s*1_ is the Michaelis constant for *y*1 for the split reaction. The steady states of *y*(2) and *y*(3) were obtained with respect to the self-replenishment cycle reaction constant *k*_*s*_ as

16 $${y}_{ss}(2)=\frac{{k}_{s}^{\ast }}{{k}_{r}}\frac{{y}_{ss}(3)}{{K}_{s3}+{y}_{ss}(3)},$$

17 $${y}_{ss}(3)=\{\begin{array}{cc}0\,(stable) & {k}_{s}^{\ast }\le \frac{{k}_{e}{k}_{r}{K}_{m2}{K}_{s3}}{{k}_{m}^{\ast }{K}_{e3}-{k}_{r}{K}_{m2}{K}_{e3}}\\ \frac{-b-\sqrt{{b}^{2}-4ac}}{2a} & \frac{{k}_{e}{k}_{r}{K}_{m2}{K}_{s3}}{{k}_{m}^{\ast }{K}_{e3}-{k}_{r}{K}_{m2}{K}_{e3}} < {k}_{s}^{\ast } < {k}_{s}^{-},{k}_{s}^{+} < {k}_{s}^{\ast }\\ NA & {k}_{s}^{-}\le {k}_{s}^{\ast }\le {k}_{s}^{+}\end{array}$$

where

18 $$\begin{array}{rcl}a & = & {k}_{m}^{\ast }{k}_{s}^{\ast }-{k}_{e}({k}_{s}^{\ast }+{k}_{r}{K}_{m2})-{k}_{s}^{\ast }({k}_{s}^{\ast }+{k}_{r}{K}_{m2})\\ b & = & {k}_{m}^{\ast }{k}_{s}^{\ast }({K}_{e3}+{K}_{s3})-{k}_{e}({k}_{r}{K}_{m2}{K}_{s3}+({k}_{s}^{\ast }+{k}_{r}{K}_{m2}){K}_{s3})-{k}_{s}^{\ast }({k}_{4}{K}_{m2}{K}_{s3}+({k}_{s}^{\ast }+{k}_{r}{K}_{m2}){K}_{e3})\\ c & = & {k}_{m}^{\ast }{k}_{s}^{\ast }{K}_{e3}{K}_{s3}-{k}_{e}{k}_{r}{K}_{m2}{{K}_{s3}}^{2}-{k}_{r}{k}_{s}^{\ast }{K}_{m2}{K}_{s3}{K}_{e3}\end{array}$$

19 $${k}_{m}^{\ast }=\frac{{k}_{m}\cdot {y}_{1}^{0}}{{K}_{m1}+{y}_{1}^{0}},\,{k}_{s}^{\ast }=\frac{{k}_{s}\cdot {y}_{1}^{0}}{{K}_{s1}+{y}_{1}^{0}}$$

20 $$\begin{array}{rcl}{k}_{s}^{-} & = & \frac{-(\,-{k}_{m}^{\ast }+{k}_{e}+{k}_{r}{K}_{m2})-\sqrt{{(-{k}_{m}^{\ast }+{k}_{e}+{k}_{r}{K}_{m2})}^{2}-4{k}_{e}{k}_{r}{K}_{m2}}}{2}\\ {k}_{s}^{+} & = & \frac{-(\,-\,{k}_{m}^{\ast }+{k}_{e}+{k}_{r}{K}_{m2})+\sqrt{{(-{k}_{m}^{\ast }+{k}_{e}+{k}_{r}{K}_{m2})}^{2}-4{k}_{e}{k}_{r}{K}_{m2}}}{2}\end{array}.$$

The fluxes of the self-replenishment and elementary cycles are given by

21 $${v}_{self}=\frac{{k}_{s}^{\ast }{y}_{ss}(3)}{{K}_{s3}+{y}_{ss}(3)}$$

22 $${v}_{elem}=\frac{{k}_{e}\cdot {y}_{ss}(3)}{{K}_{e3}+{y}_{ss}(3)}$$

In the region of *NA*, indeed, *y*(3) diverges with time; *y*(2) approaches to a limit value. By setting

$$y(3)\to \infty ,\,\frac{dy(2)}{dt}\to 0,$$

the limit of *y*(2) is given by

23 $${y}_{\mathrm{lim}}(2)=\frac{-{k}_{m}^{\ast }-{k}_{r}{K}_{23}+{k}_{e}+2{k}_{s}^{\ast }+\sqrt{{(-{k}_{m}^{\ast }-{k}_{r}{K}_{m2}+{k}_{e}+2{k}_{s}^{\ast })}^{2}+4{k}_{r}({k}_{e}+2{k}_{s}^{\ast }){K}_{m2}}}{2{k}_{r}}.$$

### Real biological models

Within a cell, self-replenishment cycles would be affected by many factors such as adjacent reactions, transcription factors, cofactors (e.g., ATP, NADH, NADPH) and allosteric metabolites. Here, I identified three real, self-replenishment cycles: the GS-GOGAT cycle, glucose Pts with glycolysis, and GX cycle in the *E. coli* central carbon and nitrogen metabolism, and mapped them on the simplified cycles used in theoretical analysis (Fig. 1), as shown in Supplementary Fig. S1. To consider the effect of such factors on the function of the three self-replenishment cycles within detailed metabolism, I numerically simulated the dynamic models of the *E. coli* central carbon and nitrogen metabolism^28,29^.

#### GS-GOGAT cycle

*E. coli* requires ammonia for synthesis of GLU and GLN, from which almost all nitrogen-containing compounds including amino acids and nucleotides are synthesized^18,30–35^. Based on detailed experimental data, Bruggeman *et al*. (2005) developed a detailed dynamic model of GS-GOGAT-GDH pathways that combine metabolic circuit with regulation by signal transduction through the covalent modification of PII and GS by UTase and ATase^28^(Table 1). Their dynamic model was employed to simulate the function of the GS-GOGAT self-replenishment cycle.

#### Glucose PTS model with glycolysis

The phosphotransferase system (PTS) is a complex group translocation system present in many bacteria^36–39^. The *E. coli* PTS transports sugars, such as glucose, mannose, and mannitol, into the cell. The first step of this reaction is phosphorylation of the substrate via phosphotransferase during transport. The glucose PTS with glycolysis forms a self-replenishment cycle, where environmental glucose reacts with phosphoenolpyruvate (PEP) to produce glucose-6-phosphate (G6P) and pyruvate (PYR) (Table 1). Kurata *et al*. developed a kinetic model of *E. coli* central carbon metabolism with glucose PTS, which reproduced the experimental data obtained from several knockout mutants^29,40,41^. This model was used to analyze how the self-replenishment cycle of the glucose PTS uptakes environmental glucose^40^.

#### TCA and GX cycles

The glyoxylate cycle, identified by Kornberg *et al*.^42^, provides a simple and efficient strategy for converting acetyl-CoA into anaplerotic and gluconeogenic compounds when complex sources such as glucose are not available^43^. If some intermediate metabolites of the TCA cycle are degraded or removed, the TCA cycle flux cannot be maintained without any anaplerotic (phosphoenolpyruvate carboxylase (Ppc)) reactions. In a Ppc knockout mutant of *E. coli*, however, OAA seemed to be supplied to drive the TCA cycle^44,45^. Intensive analysis suggested that the GX cycle forms a self-replenishment cycle (Table 1) and can supply OAA instead of the Ppc reaction. The kinetic model of *E. coli* central carbon metabolism was numerically simulated^40^ to analyze how the self-replenishment cycle of the GX cycle functions as an anaplerotic reaction.

### Numerical simulation

All numerical simulations were carried on Matlab (version 2019a, The MathWorks, Inc). The simulation programs of the self-replenishment cycle, elementary cycle, and self-replenishment and elementary cycles are registered in the BioFNet database^4^ as IDs of 372, 374 and 375, respectively, and presented as Supplementary Information. The program of the kinetic model for *E. coli* central carbon metabolism is freely available at the CADLIVE site (http://www.cadlive.jp/cadlive_main/Softwares/KineticModel/Ecolimetabolism.html).

## Results

### Self-replenishment cycle

**Figure 2:**
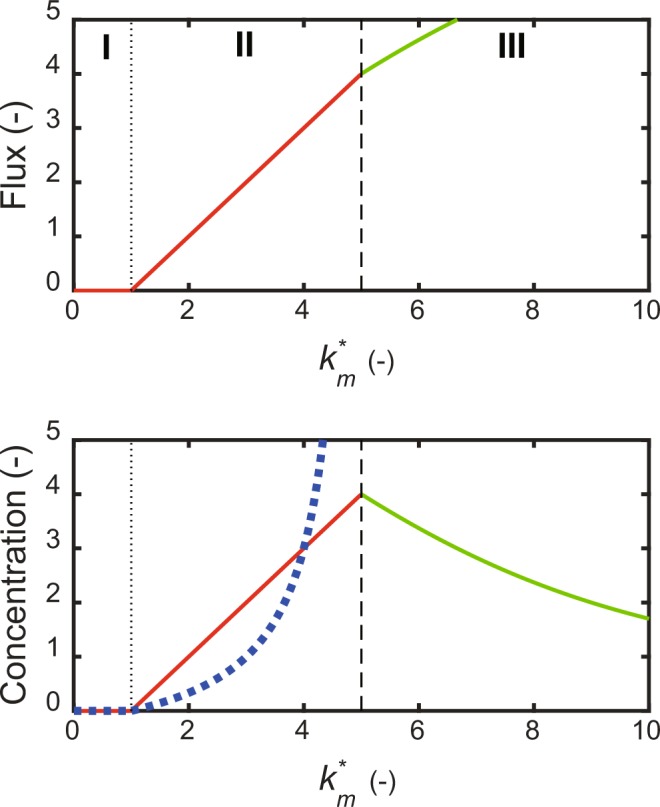
Threshold response of the self-replenishment cycle. The steady-state and limit values of *y*(2) and *y*(3), and the self-replenishment cycle flux were plotted with respect to *k*_m_*. *K*_*m*2_ = 1, *K*_*s*_ = 4, *K*_*s*3_ = 1, *K*_*r*_ = 1. The broken vertical line is the discontinuity point; the dotted vertical line indicates derivative discontinuity point. Each diagram is divided into three regions (I, II, and III) according to *k*_m_*. I and II are the stable regions; III is the unsteady-state region. Flux panel: The red, solid line is the steady-state self-replenishment cycle flux; the green, solid line is the limit value of the flux. Concentration panel: The red, solid line is the steady-state concentration of *y*(2); the blue, dotted line is the steady-state concentration of *y*(3); the green, solid line is the limit value of *y*(2), while *y*(3) that diverges in III is not plotted.

To reveal a distinct function of the self-replenishment cycle (Fig. 1a), Eqs (1, 2) were solved for the steady state levels or limit values of *y*(2), *y*(3), and *v*_*self*_. The solutions, given by Eqs. (3–6), were the threshold response for input, merge rate constant *k*_m_* (Eq. (5)) (Fig. 2). The time course of three molecules are shown in Supplementary Fig. S2. The solutions were classified into three regions (I, II, and III) by the lower derivative discontinuity point (${k}_{m}^{\ast }={k}_{r}{K}_{m2}$) and the upper discontinuity point (${k}_{m}^{\ast }={k}_{s}+{k}_{r}{K}_{m2}$). The steady-state levels of *y*(2), *y*(3), and *v*_*self*_ were stable zero in region I. The steady-state levels of *y*(2), *y*(3), and *v*_*self*_ steeply increased in region II. Molecule *y*(2) presented threshold response to specific parameter *k*_m_*. The threshold value of *k*_m_* was given by the derivative discontinuity point as follows.

24 $$threshold\_{k}_{m}^{\ast }={k}_{r}{K}_{m2}$$

The threshold value increased with an increase in the removal rate of *y*(2) and with a decrease in the synthesis rate of *y*(2), determined by the balance between the removal and synthesis rates. The existence of the zero region (region I) demonstrated that the self-replenishment cycle generates a play or clearance to switch on the cycle. In region III, they had no positive, steady-state solution, because the accumulator function surpassed the removal one. Indeed, *y*(3) diverged with time, while *y*(2) approached the limit value given by Eq. (7) and the flux also did.

### Elementary cycle

**Figure 3:**
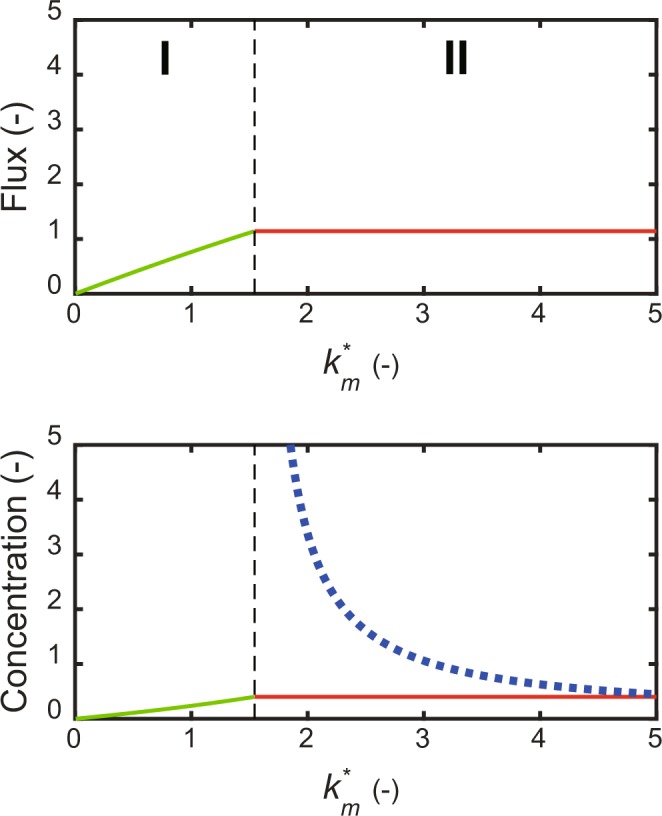
Smooth response of the elementary cycle. The steady-state solutions of *y*(2) and *y*(3), and the elementary cycle flux were plotted with respect to *k*_m_*. *K*_*m*2_ = 1, *K*_*e*_ = 4, *K*_*e*3_ = 1, *K*_*r*_ = 1, *v*_a_ = 0.4. The broken vertical line is the discontinuity point. Each diagram is divided into two regions (I and II) according to *k*_m_*. I is the stable, steady-state region; II is the unsteady-state region. Flux panel: The red, solid line is the steady-state cycle flux; the green, solid line is the limit value of the flux. Concentration panel: The red, solid line is the steady-state concentration of *y*(3); the blue, dotted line is the steady-state concentration of *y*(2); the green, solid line is the limit value of *y*(3), while *y*(2) that diverges in I is not plotted.

To investigate the function of the elementary cycle (Fig. 1b), Eqs (8, 9) were solved for the steady state levels and limit values of *y*(2), *y*(3), and *v*_*elem*_. The solutions, given by Eqs. (10–12), are depicted as shown in Fig. 3. They were classified into two regions (I and II) by the discontinuity point. Differing from the self-replenishment cycle, they had no derivative discontinuity point with respect to input rate constant *k*_m_*, indicating that no threshold exists. In region I, they had no positive, steady-state solution, because *y*(2) accumulated due to a low input reaction rate. Indeed, *y*(2) diverged with time, while *y*(3) approached the limit value given by Eq. (13) and the flux also did. In region II, the steady sate of *y*(3), given by Eq. (11), was the constant that were determined by the balance between the replenishment flux and removal flux.

### Self-replenishment and elementary cycles

**Figure 4:**
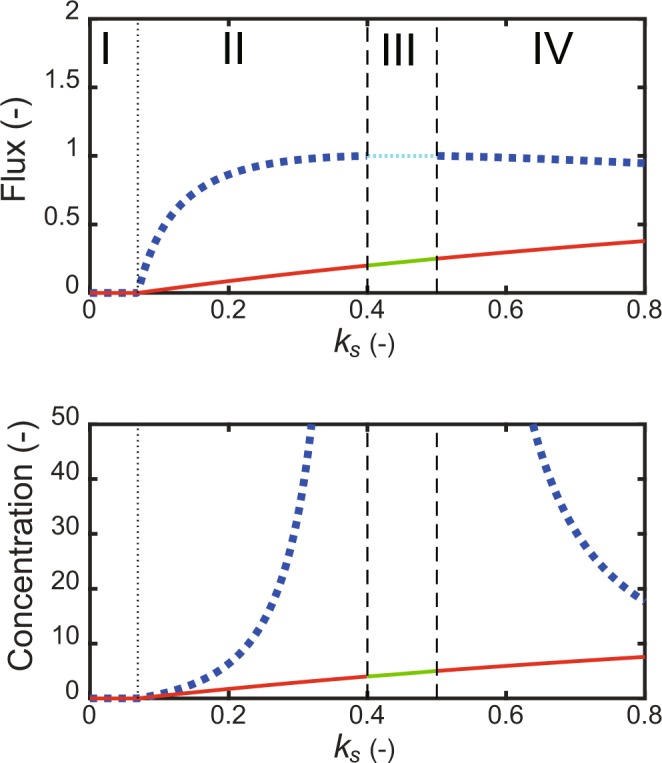
Threshold response of self-replenishment and elementary cycles. The steady-state and limit values of *y*(2) and *y*(3), and the fluxes of the combined cycles were plotted with respect to and the self-replenishment cycle rate constant *k*_*s*_. ${k}_{m}^{\ast }=1.5$, *K*_*m*2_ = 1, *K*_*s*1_ = 1, *K*_*s*3_ = 1, *K*_*e*_ = 1, *K*_*e*3_ = 1, *K*_*r*_ = 0.05. The broken vertical line is the discontinuity point; the dotted vertical line is the derivative discontinuity point. The diagram is divided into four regions (I, II, III, and IV) according to *k*_*s*_. I, II, and IV are the stable, steady-state regions; III is the unsteady-state region. Flux panel: The red, solid line is the steady-state self-replenishment cycle flux; the blue, dotted line is the steady-state elementary cycle flux; the green, solid line is the limit value of the self-replenishment cycle flux; the cyan, dotted line is the limit value of the elementary cycle flux. Concentration panel: The red, solid line is the steady-state concentration of *y*(2); the blue, dotted line is the steady-state concentration of *y*(3); the green, solid line is the limit value of *y*(2), while *y*(3) that diverges in III is not plotted.

The elementary cycle typically stops without any anaplerotic reaction, when a removal reaction occurs (Fig. 1b, *v*_*a*_ = 0, *v*_*r*_ > 0). Since the self-replenishment and elementary cycles (Fig. 1c) correspond to the simplified GX and TCA cycles, respectively, I investigate how the self-replenishment cycle drives the elementary cycle. To analyze the effect of the self-replenishment cycle rate constant *k*_*s*_ on the elementary cycle, Eqs (14, 15) were solved for the steady-state values of *y*(2), *y*(3), and the fluxes of both the cycles. The solutions are given by Eqs (16–22). When *b*^2^−4*ac* > 0 (Eq. (17)), the steady-state solutions presented the threshold response of *k*_*s*_ and were divided into four regions (I, II, III, and IV) by the three critical points (Fig. 4). The first point was the derivative discontinuity obtained by setting *c* = 0 (Eq. (18)). The second and third points were the discontinuity points obtained by setting *a* → 0 (Eq. (18)). The steady-state concentrations of *y*(2) and *y*(3) and the steady-state fluxes of both the cycles were stable zero in region I, because the removal reaction surpassed the self-replenishment cycle-based accumulation reaction. They simultaneously increased in region II, showing a threshold response, where both the removal and accumulation were balanced. It demonstrates that the self-replenishment cycle works as the anaplerotic reaction to run the elementary cycle. Region III had no positive, steady-state solution, where *y*(3) diverged; *y*(2) approached the limit value given by Eq. (23). Both the fluxes approached the limit values. In region IV, *y*(2), *y*(3), and the fluxes of both the cycles returned to the steady-state level again, because a large value of *k*_*s*_ decreased *y*(3). The steady state level of *y*(3) decreased with an increase in *k*_*s*_; *y*(2) gently increased. The first critical point or the threshold value of *k*_*s*_ was given by

25 $$threshold\_{k}_{s}=\frac{{k}_{e}{k}_{r}{K}_{m2}{K}_{s3}}{{K}_{e3}({k}_{m}^{\ast }-{k}_{r}{K}_{m2})}\frac{{K}_{{\rm{s1}}}+{y}_{1}^{0}}{{y}_{1}^{0}}.$$

The existence of a zero concentration/flux region showed that the self-replenishment cycle generates a play or clearance to switch on both the cycles. The threshold value was determined mainly by the elementary cycle with *k*_*e*_ and the removal reaction with *k*_*r*_. An increase in removal rate constant *k*_*r*_ increased the threshold value. An increase in rate constant *k*_*e*_ increased the threshold value, because the elementary cycle took *y*(3) from the self-replenishment cycle, decreasing the flux of it.

### GS-GOGAT cycle

**Figure 5:**
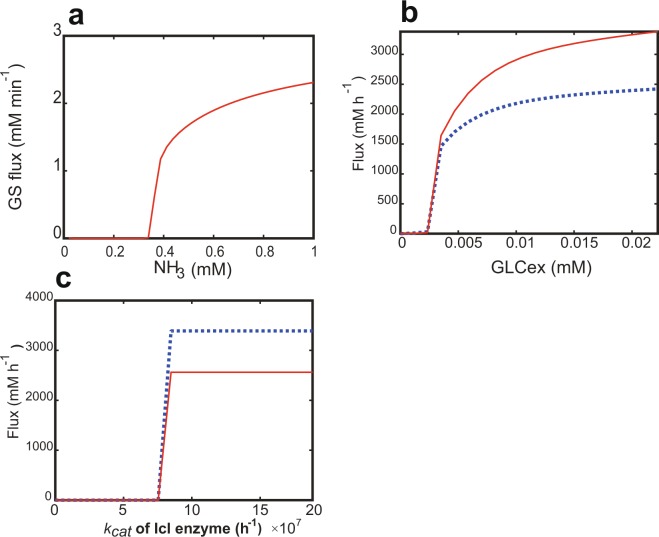
Digital switch-like responses in realistic, detailed kinetic models of *E. coli*. (**a**) GS-GOGAT cycle. The GX flux was simulated for 1,000 min, while changing the environmental ammonium concentration (constant). The final flux was plotted with respect to the ammonium concentration. (**b**) Glucose PTS with glycolysis. The PTS flux and enolase (Eno) flux were simulated for 5 h, while changing the environmental glucose concentration (GLCex) (constant). Their final fluxes were plotted with respect to the glucose concentration. The red, solid line is the Eno flux; the blue, dotted line is the glucose uptake flux. (**c**) TCA and GX cycles. The TCA flux and GX flux were simulated for 5 h, while changing *k*_*cat*_ of the Icl enzyme. Both the fluxes at 5 h were plotted with respect to the *k*_*cat*_ of the Icl enzyme. The red, solid line is the GX flux; the blue, dotted line is the TCA flux.

A detailed kinetic model of the ammonia assimilation system, developed by Brugemann *et al*.^28^, was simulated to investigate how the GS-GOGAT cycle is implemented into real biochemical networks. The GS flux was simulated for 1,000 min, while changing the environmental ammonium concentration (constant variable) (Fig. 5a). Since ammonia concentration corresponds to *y*(1) of *k*_m_*, the simulation result can be compared to the theoretical analysis (Eqs (3–5)). The play region existed in the region from zero to a threshold point of 0.33 mM ammonium, where the flux was zero. The GS flux showed a linear rise in a relatively low concentration of ammonia.

### Glucose Pts with glycolysis

To investigate the function of glucose PTS with glycolysis, a detailed kinetic model of the *E. coli* central carbon metabolic system was simulated with respect to a change in environmental glucose concentration^40^. Since glucose concentration corresponds to *y*(1) of *k*_m_*, the simulation result can be compared to the theoretical analysis (Eqs (3–5)). The environmental glucose concentration was set to a constant variable. The glucose uptake flux and enolase (Eno) flux by the glucose PTS on 5 h were plotted with respect to the environmental glucose concentration (Fig. 5b). The Eno flux was chosen as an indicator of glycolysis metabolism. The Eno flux showed a threshold response, with a threshold point of 0.0023 mM environmental glucose. The cells did not uptake glucose at less than the threshold. At more than the threshold, the glucose uptake and glycolysis fluxes steeply increased to a very high level. Both the flux showed a linear rise in a relatively low concentration of glucose.

### GX and TCA cycles

To demonstrate the mechanism by which the GX cycle supplies OAA to drive the TCA cycle in the Ppc knockout mutant, a detailed kinetic model of the Ppc knockout mutant was numerically simulated in a batch culture^40^. In this simulation, the fluxes of the TCA and GX cycles were calculated for 5 h with respect to a change in *k*_*cat*_ of the isocitrate lyase (Icl) or malate synthase (MS) enzymes, directly responsible for the GX cycle (Fig. 5c), which corresponds to kinetic parameter *ks* in Eqs (14, 15). A critical or threshold point exists that remarkably shifts the metabolic status. At an Icl *k*_*cat*_ value of less than the threshold point, the GX and TCA fluxes were stable zero, showing the play region. Above the threshold, both the fluxes linearly shifted to a high level, supplying OAA to drive the TCA cycle. This simulation result agreed to the experimental data that the GX and TCA cycles greatly flow at high Icl/Ms expressions in the Ppc knockout mutant, while the GX cycle stops in low Icl/Ms expressions^45^. This demonstrates that the GX cycle works as the self-replenishment cycle to produce the threshold response and as anaplerotic reaction to drive the TCA cycle in the *E. coli* central carbon metabolism. Interestingly, this response is an all or nothing response or a digital switch behavior.

## Discussion

To date, many scientists have investigated such an amplifier function of different metabolic cycles^3,5,10,12,14,17^, but they have overlooked or missed the switching function of the self-replenishment cycle that doubles the products and recycles them as substrates. This study first demonstrated a design principle that the self-replenishment cycle generates a threshold response and provides a play region to initiate the cycle reaction, differing from the well-known elementary cycles that show a smooth response.

The *E. coli* GS-GOGAT cycle reaction did not occur until the ammonium concentration exceeded a threshold value of 0.33 mM, suggesting that it avoids uptake of environmental ammonium of less than the threshold value. In the same manner, the glucose PTS avoided consumption of environmental glucose at less than 0.0023 mM. The threshold value was very low, because *E. coli* typically grows in a batch culture with 20 mM glucose. The threshold value is too small to measure *in vivo* and *in vitro*. This may be the reason why this threshold response has received little attention before. The self-replenishment cycles can be used to implement a strategy of shutting down the uptake of a low concentration of substrate.

The TCA cycle is a hub of central metabolism of both energy production and biosynthesis and is directly involved in anaplerotic and cataplerotic reactions. In *E. coli*, OAA is typically supplied by an anaplerotic reaction (Ppc) to drive the TCA cycle. This raises a question of how the Ppc knockout mutant drives the TCA cycle. Previous experimental studies suggested that the GX cycle supplied OAA to the TCA cycle^44,45^. This study simulated the detailed kinetic model of the Ppc knockout mutant^40^ to demonstrate that the GX cycle works as an anaplerotic reaction to drive the TCA cycle (Fig. 5c). To theoretically confirm such a function, I simplified them into the self-replenishment and elementary cycle model (Fig. 4). The simplified GX and TCA cycles simultaneously increased after the threshold, where the GX cycle supplied the substrate to run the TCA cycle. In other words, the TCA cycle did not occur until the GX cycle accumulated the recycled product or substrate. The threshold response of the GX and TCA cycles is a distinguished feature from the smooth response of the typical Ppc anaplerotic reaction to the TCA cycle (*v*_*a*_ in Eq. (8)). The smooth response is readily estimated by Eq. (11), where the product concentration is linear to the external replenishment flux. Interestingly, the detailed kinetic model of the Ppc knockout mutant (Fig. 5c) showed much more steep responses than the theoretical model (Fig. 4), i.e., it provided two distinct metabolic states in a digital switch manner with respect to the enzyme activity within the GX cycle.

Different types of signal sensors or switches have been extensively investigated in gene regulatory networks and signal transduction pathways. Digital switch-like responses are known to be generated by specific mechanisms such as cooperativity and bistability^46,47^. Cooperativity provides ultrasensitivity to an enzyme reaction and gene expression in the form of the Hill equation. A high Hill coefficient presents a sigmoidal response or a digital switch-like behavior. Bistability caused by positive feedback loops, such as mutual activation/repression and positive autoregulation^46,47^, are critically responsible for a digital switch-like response showing two distinct output levels. Importantly, the digital-like response of the self-replenishment cycles neither involves the bistability mechanism nor cooperativity. To intelligibly understand its design principle, the self-replenishment cycle (Fig. 1a) was further simplified into a two-component model:

$${{\rm{y}}}_{1}+{{\rm{y}}}_{2}- > 2{y}_{2}.$$

Assuming that the concentration of y_1_ is constant, the equation is given by:

26 $$\frac{dy(2)}{dt}={k}_{m}\frac{y(2)}{{K}_{m}+y(2)}-{k}_{r}\cdot y(2)$$

where y(2) is the concentration of y_2_, *k*_*m*_ and *K*_*m*_ are the kinetic constants. The steady-state solution of y(2) is given by

27 $${y}_{ss}(2)=\{\begin{array}{cl}0 & a\le {K}_{m}\\ a-{K}_{m} & a > {K}_{m}\end{array}$$

where

28 $$a=\frac{{k}_{m}}{{k}_{r}}$$

This shows a ReLU or threshold response with respect to α (Supplementary Fig. S3), demonstrating that self-activation or a positive feedback loop is a basic mechanism responsible for the threshold response.

The realistic, detailed kinetic model showed a much steeper response than the theoretical models (Fig. 5c). I considered the mechanism by which the detailed model shows such steep changes. The theoretical models described the removal reactions in the form of the first order reaction so that the equations can be analytically solved. On the other hand, the realistic, detailed kinetic model described the removal reactions in the form of the Michaelis-Menten type or nonlinear equations. To investigate how the kinetics types of the removal reactions alter the threshold response, the first-order removal reaction of Eq. (26) is replaced by the Michaelis-Menten type equation as follows:

29 $$\frac{dy(2)}{dt}=a\frac{y(2)}{{K}_{m}+y(2)}-\frac{y(2)}{{K}_{d}+y(2)}$$

where *K*_*d*_ is the dissociation constant. At *K*_*m*_ = *K*_*d*_ and *t* → ∞, it is given by:

30 $${y}_{\infty }(2)=\{\begin{array}{ll}0 & a < 1\\ {y}_{0} & a=1\\ \infty  & a > 1\end{array}$$

where *y*_0_ is the initial concentration of y_2_. It shows all or nothing response (Supplementary Fig. S3). Use of the Michaelis-Menten type equation was demonstrated to make the rise much steeper. Actually, in the realistic, detailed models, each metabolic concentration never diverges, because the metabolic fluxes and metabolite concentrations are bounded or constrained by other reactions. The distinct existence of the upper flux limit (Fig. 5c) is caused by the fact that the total input flux to the TCA and GX cycles is bounded and their intermediate metabolites are removed from the cycles as cataplerotic reactions.

I discuss the proposed threshold response in relation to ReLU. In general, a linear input-output relationship was generated by a specific type of negative feedback and negative regulation and by a specific enzyme reaction with the conserved total concentration of inhibitor and substrates^27,48,49^. While the self-replenishment cycle would have no such distinct mechanisms responsible for linear function, it can linearly respond to substrate above a threshold when ${y}_{0}^{1}$ is less than *K*_*m1*_, as suggested by Eqs (3, 5). It also presents a linear response in a limited region of a low substrate concentration, as shown in Fig. 5, when the uptake reaction of substrate follows a Michaelis-Menten equation. In summary, the linearity of the self-replenishment cycle depends on the types of kinetics.

The prediction of the threshold response (Fig. 5) would be experimentally validated as follows. One measures the uptake fluxes of environmental glucose and ammonia while changing their concentrations. Since a threshold concentration is supposed to be very low, predicted by the numerical simulation (Fig. 5a,b), a high-resolution instrument is required that measures such a low threshold value. The GX cycle was experimentally demonstrated to stop in wild type with the Ppc anaplerotic reaction, while it greatly flowed in the *ppc* (encoding anaplerotic reaction) knockout mutant^44,45^, where the activities of Icl/Ms enzymes were increased. To demonstrate the digital switch-like response of the TCA and GX cycles (Fig. 5c), one measures their fluxes in the *ppc* knockout mutant, while changing the expression levels of the *icl*/*ms* genes. In such experiments, one uses the icl/ms genes fused to appropriate inducible promoters, after deleting their original genes.

The threshold response is useful in biological functions that should ignore low levels of inputs and respond over a wide range of them. The threshold response has a desirable property to present a distinct threshold that separates a non-nutrient uptake state from a nutrient uptake one^27^. It can be also regarded as a noise filter, where a cell does not uptake a nutrient below a certain level. Since the nutrient uptake system generally requires energy cost, it may evolve so as to consider the balance between the advantage of acquiring nutrients for cell growth and burden of its related energy cost. The GS-GOGAT cycle consumes energy (ATP) and reducing powers (NADPH) for each turn of cycles. The glucose Pts with glycolysis consumes PEP with a high energy phosphate bond, although the glycolysis pathway generates ATP and NADH. Those cycles may evolve as the nutrient uptake regulator that can completely stop the uptake of a very low concentration of ammonia or glucose to avoid wasting energy or reducing power. On the other hand, the GX cycle evolves as the internal anaplerotic pathway, which is formed by connecting the three metabolites of AcCoA, OAA and ICIT within the TCA cycle (Supplementary Fig. S1), to drive the TCA cycle in the absence of the external anaplerotic reaction. Since the GX cycle does not require any energy cost but generates reducing power, it may evolve in a different way from the above two nutrient uptake cycles.

## Supplementary information

Supplementary figures

Supplementary figures
