# Supplementary material for: Self-replenishment cycles generate a threshold response
Source: Sci Rep. 2019 Nov 20;9:17139. doi: 10.1038/s41598-019-53589-1 (PMC6868230; doi:10.1038/s41598-019-53589-1)
Supplement: Supplementary file 1 — Supplementary figures [file 41598_2019_53589_MOESM1_ESM.pdf]

Supplemental information

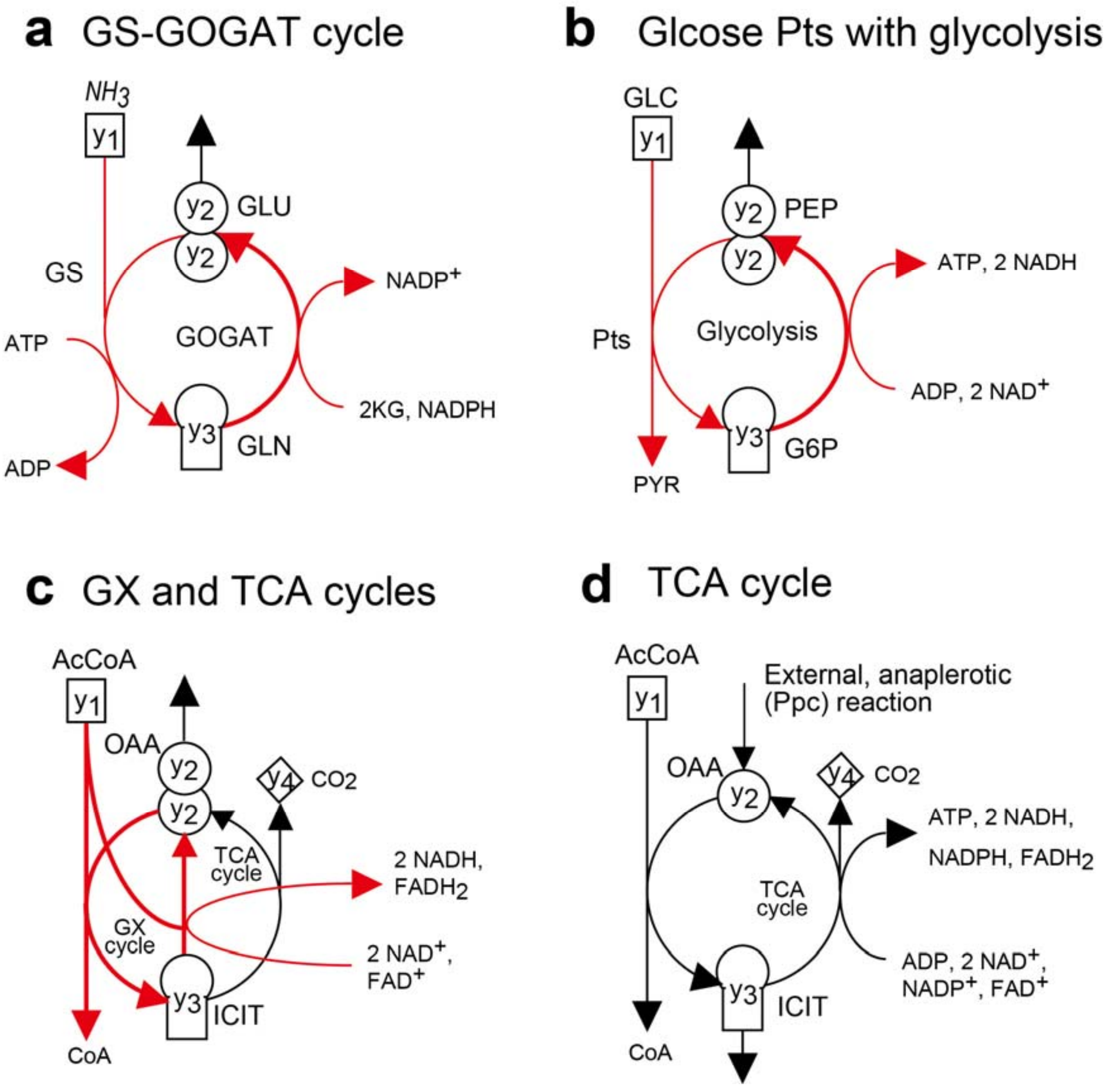

Figure S1 Biochemical network map of three biological self-replenishment cycles

The biological cycles are mapped on the simplified cycles used in theoretical analysis (Fig. 1). **a**: GS-GOGAT cycle. **b**: Glucose Pts with glycolysis. **c**: GX and TCA cycles. **d**: TCA cycle (elementary cycle with an external reaction). The self-replenishment cycles are colored in red. Derivation of the cycle pathways is described in Table 1.

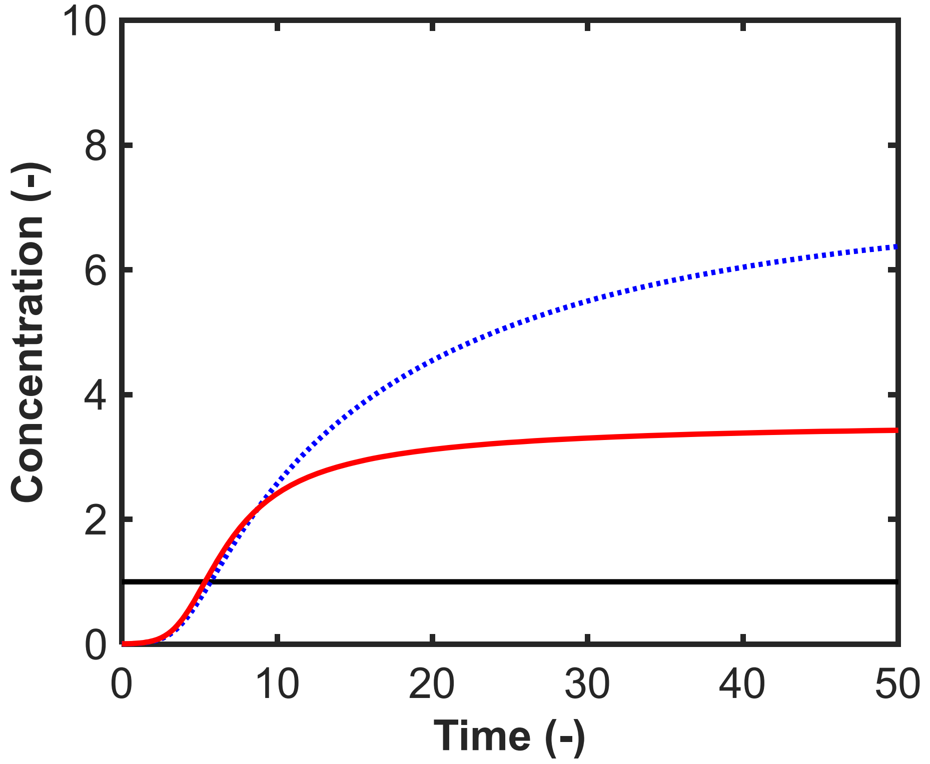

Figure S2 Dynamic simulation of the self-replenishment cycle.

The black line, the red line, and the blue dotted line are the time courses of $y(1)$, $y(2)$ and $y(3)$, respectively. k_m* is set to 4.5, where all the concentrations approach to the steady state. $K_{m2} = 1$, $k_s = 4$, $K_{s3} = 1$, $k_r = 1$. The initial concentrations of $y(1)$, $y(2)$ and $y(3)$ are set as follows: $y_1^0 = 1$, $y_2^0 = 0.01$, $y_3^0 = 0$.

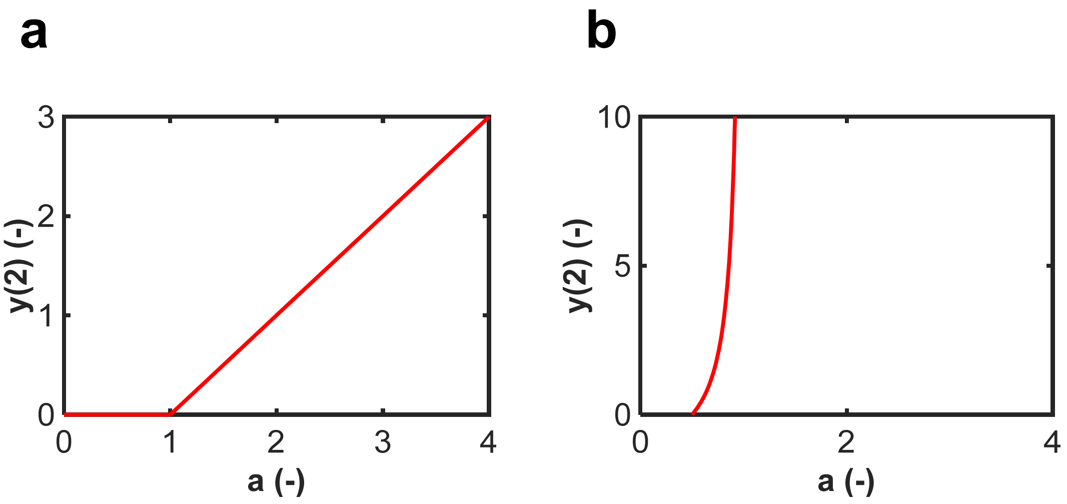

Figure S3 ReLU and digital switch-like responses of the simplified self-replenishment cycle model

The steady-state level of y was plotted with respect to constant a.

a: Linear degradation model ($K = 1$).

b: Degradation model with Michaelis-Menten type equation ($K_m = 1$, $K_d = 1$). The dynamics were simulated for $t = 10{,}000$; the final concentration was plotted.

Table S1 Examples of complex cycles

Name	Reaction	Comment
Double substrate cycle	(Fist cycle)<br>PEP + **X** -> PYR + **Y**<br>G6P + **Y** -> GLC + **X**<br>(Second cycle)<br>**PYR** -> LAC<br>LAC -> **PYR**	#1
Coenzyme recycling cascade with coenzyme conservation	GAP + **NAD^+** -> PYR + NADH<br>PYR + NADH -> LAC + **NAD^+**	#2
Coenzyme recycling cascade with turbo design	GLC + 2 **ATP** -> FBP + 2 ADP<br>FBP + 4 ADP ---> 2 PYR + 4 **ATP**	#3

Bold compounds are the substrates/products responsible for forming the cycle.

"--->" indicates multiple reactions are simplified.

#1 The first cycle supplies PYR to the second (PYR-LAC) cycle, which accelerates the second cycle.

#2 In the GAP-PYR-LAC reaction cascade, the total cofactor (NAD^+ + NADH) concentration is conserved.

#3 In the GLC-FBP-PYR reaction cascade, externally supplied ADP increases the total cofactor (ADP + ATP), which accelerates the cascade reaction.
